# Morphological awareness in developmental dyslexia: Playing with nonwords in a morphologically rich language

**DOI:** 10.1371/journal.pone.0276643

**Published:** 2022-11-17

**Authors:** Chiara Melloni, Maria Vender

**Affiliations:** Department of Cultures and Civilizations, University of Verona, Verona, Italy; University of Ljubljana, SLOVENIA

## Abstract

Although phonological deficits are unanimously recognized as one of the key manifestations of developmental dyslexia, a growing body of research has reported impairments in morphological abilities. Our study aimed at casting further light on this domain by investigating the morphological awareness skills of 21 children with dyslexia (mean age 9.10 years old) and 24 children with typical development (mean age 10.3 years old). All children were monolingual speakers of Italian, which is a morphologically rich language characterized by complex inflectional and derivational paradigms. We developed an experimental protocol inspired by Berko’s Wug test and composed of 11 tasks addressing inflectional and derivational processes. Participants were asked to manipulate nonwords of various lexical categories, modeled after the phonotactic structure of Italian, and manipulation involved both word formation and base retrieval. Conditions of the experiments were based on verb conjugation classes differing in frequency, productivity, regularity, and formal transparency. Results confirmed that morphological skills are impaired in dyslexic children, who performed significantly more poorly than their age-matched peers in all tasks. Children with dyslexia were especially challenged by tasks and conditions requiring advanced morphological awareness skills, such as the retrieval of infinitives of infrequent and irregular conjugation classes. The educational and clinical implications of these findings are discussed.

## 1. Introduction

Morphological awareness refers to the understanding of the structure of words as combinations of smaller meaningful units, known as morphemes, and to the ability to manipulate them [1]. A growing body of research has highlighted the crucial role of morphological awareness in the development of reading skills in alphabetic writing systems [2–8]. Findings indicated that morphological skills uniquely predict reading achievements, especially at later stages of literacy acquisition. This predictive role has not only been found in English or French, i.e., languages with an opaque writing system where, due to a highly irregular phoneme-to-grapheme mapping, morpheme recognition is expected to aid reading processing, both in terms of accuracy and fluency. Crucially, morphological skills also emerged as a good predictor of reading proficiency in languages with more transparent orthographies (see [9] on Portuguese and [10] on Greek), playing a key role in reading fluency (see [11] on Italian).

Given its role in reading acquisition, research has also considered morphological awareness in reading-impaired individuals, especially in children with developmental dyslexia. Although dyslexia is characterized by major phonological deficits, difficulties go beyond phonology and also affect syntactic and morphological skills [12–14]. Interestingly, however, research has found that morphological awareness, prompting the identification of strings of letters corresponding to morphemes, is exploited to compensate for the spelling and reading deficits characterizing dyslexia [15, 16].

Although the number of studies on morphological skills in dyslexia is growing, research has still to address several issues and classes of data, especially in languages with rich morphology, and novel experimental evidence could lead to a better understanding of morphological awareness across different languages and populations. For instance, research on morphological awareness in dyslexia has considered a variety of morphological variables and measures but, to the best of our knowledge, the role of formal transparency, frequency, and productivity of verbal paradigms across different types of word formation operations has not been previously investigated in detail.

The current study aims to explore morphological awareness skills in Italian-speaking children with dyslexia and with typical development. Italian is a fusional language, characterized by complex inflectional and derivational paradigms, and research on morphological abilities in Italian dyslexic children is still scarce. To compare the profiles of reading impaired and typically developing children in the challenging domain of Italian morphology, we designed a novel protocol comprising tasks of inflection and derivation, considering both the production of complex words and the retrieval of base forms, and with conditions entailing different levels of morphological awareness, as we will discuss more in detail below.

### 1.1. Morphological knowledge, morphological awareness, and reading achievements

Morphology plays a central role in language acquisition and children start to experiment with morphemes and create new words and word forms very early, around 18 months of age and usually by their first year of speech [17]. Although children start to learn morphology early on, its acquisition and full mastery is a gradual and multifaceted process, with different aspects of morphology developing at different rates and times [18]. The earliest manipulations usually concern the expression of inflectional features (such as gender and number) while consistent use of derivational forms emerges at an older age since derivation implies the identification of affixal meanings. This is a process that can be especially taxing in the case of unproductive and/or semantically and formally opaque derivational rules [19]. Indeed, many variables influence the rapidity and accuracy of children’s morphological acquisition and processing: for instance, transparency, i.e., correspondence between a feature’s value and a formal exponent, facilitates the acquisition of inflectional features (see [20] on the acquisition of gender in Russian and Bulgarian).

The morphological skills acquired by children in the first years of life roughly correspond to an “implicit knowledge organization” [21], i.e., a form of epilinguistic, unconscious knowledge that develops spontaneously. These abilities are a necessary basis for the development of morphological awareness in later years. Specifically, according to [22]’s framework, morphological awareness is part of metalinguistic knowledge and, as such, involves conscious reflection upon word structure, while skills of monitoring and planning are required for the deliberate manipulation of morphemic units. Whilst epilinguistic abilities are acquired spontaneously, morphological awareness is crucially related to schooling and literacy acquisition [10, 23]. Therefore, although a low level of morphological awareness can be found in preschoolers [24], a more refined awareness of morphemic structures chiefly develops when explicit instruction about word composition and growing experience in reading trigger an open reflection upon morphemic structure in complex words. Crucially, not all morphological awareness skills develop simultaneously, but their emergence seems tied to the complexity and frequency of the systems and their utility in further tasks [21]. In particular, awareness of inflectional morphology is usually detected earlier (in the first school years) than awareness of derivational morphology, which emerges in the fourth grade and continues to develop afterward (see [10] for an overview).

Given its role in literacy acquisition, experimental research has increasingly targeted morphological awareness across several languages and populations in recent years. Common measures of morphological awareness are production tasks, where the child is asked to complete a sentence with a target word as in e.g., “Teach. My sister tells me that I am a good *teacher*” and decomposition tasks or, more specifically, base retrieval tasks as in e.g., “Driver. Children are too young to *drive*” (see [2, 4, 10, 25–27]). In both production and decomposition tasks, children must give an explicit response tapping conscious awareness of the target morpheme and both types of tasks have been found highly reliable for predicting reading success [28]. Other production and decomposition tasks have deployed nonce words, rather than real words, following the ingenious design of Berko’s Wug test [24], which abstracts away from the subject’s vocabulary knowledge, while tapping more directly morphological abilities such as applying inflectional and derivational patterns to novel stems (i.e., to make the plural of the nonce word *wug*, children cannot rely on a memorized plural form, but have to resort to the application of a rule or schema of plural inflection).

These tasks have been mostly employed to measure children’s morphological awareness and, in many studies, its correlation to reading skills. Moreover, the effects of morphological awareness on reading achievement have also been studied in training programs aimed at reinforcing morphological skills (see [29, 30] for comprehensive reviews). Research in this area has seen an upsurge since the nineties [31, 32] and shows that children trained to identify and manipulate morphemes and to reflect upon the morphosemantic relations among complex lexical items are those who perform better in reading and spelling tasks, with positive effects at any age. Findings on the positive influence of morphological instruction are particularly important for children with poor phonological skills, such as children with developmental dyslexia or language impairments. Indeed, readers who cannot rely on solid (meta-)phonological skills are more prone to resort to morphemic awareness as a compensatory strategy for overcoming their reading and spelling deficits. In these cases, in particular, dedicated training programs aimed at developing morphological awareness skills could allow children to compensate for their grapheme-to-phoneme mapping deficits by relying more perspicuously on morphological cues and resulting in better word reading fluency, accuracy, and comprehension. This could be the case for children affected by developmental dyslexia, as discussed in the next section.

### 1.2. Morphological awareness and developmental dyslexia

As indicated in the DSM-5 [33], Developmental Dyslexia (dyslexia henceforth) belongs to the overarching category of specific learning disorders, defined as neurodevelopmental disorders that impede a person’s ability to learn and use specific academic skills, such as reading, writing, and arithmetic, which serve as the foundation for most other academic learning. The main behavioral manifestations of dyslexia are deficits in learning to read and spell, which are especially marked in languages with deep orthographic systems, like English and French. In languages with shallow orthographies like Italian or Greek, impairments of people with dyslexia are less manifest since grapheme-phoneme conversion rules are more consistent and easier to learn than in deep orthographies. Since phonological abilities are crucial for learning to read in alphabetic systems, it comes as no surprise that dyslexia is mainly characterized by a phonological deficit [34–39]. It remains to be established whether the difficulties experienced by dyslexics in other domains, such as vocabulary development [40–42] and grammatical abilities [43–47] may be derived from the core phonological deficit or are the expression of impairments extended to other language domains beyond phonology, or if they derive from an impaired cognitive profile also characterized by poor (verbal) working memory and executive functions [12, 48].

Besides extensive phonological deficits, children with dyslexia were found impaired in morphological abilities too, as reported in several studies [13, 14, 21, 49, 50]. Inflectional morphology is one of the best-studied domains. In [13], children with dyslexia underperformed age-matched controls on a Wug test assessing children’s ability to apply past tense agreement and pluralization rules to nonce words. Similar results were found by [49], testing gender and number agreement on sentence completion tasks where children were asked to identify target forms on the basis of gender and number features. Children with dyslexia had the worst performance in all tasks in comparison with chronological age-matched controls and even with reading age-matched controls [21, 51].

Impairments in the morphological domain have also been reported in children with dyslexia learning languages with rich morphology and shallow orthographic system, such as Bosnian, Greek, and Italian. In a study on Bosnian, [52] found that dyslexic children performed significantly worse than age-matched controls across several inflection and derivation tasks, especially in the production of suffixed words, declension of personal pronouns, and gender-based tasks. A recent study on Greek [53] found that children with dyslexia in third grade have impairments in inflectional awareness and vocabulary in comparison to their age-matched peers. Morphological awareness deficits in Greek dyslexic children were also found by [54], exploring inflection, derivation, and compounding. Moreover, in a study on Italian, [14] reported the findings of a Wug test aimed at measuring the subjects’ ability to apply pluralization rules to nonwords in the morphologically complex context of Italian nominal inflection. Results have shown that children with dyslexia display poorer morphological skills in comparison to age-matched controls, showing lower accuracy in the task. Moreover, the children’s performance in this task was significantly related to their reading proficiency and could predict accuracy in word reading independently of phonological awareness and working memory. Differently from other research assessing morphological skills, [14]’s study has identified deficits in morphological awareness that cannot depend on lexical knowledge limitations, as the use of nonwords allows to identify the subject’s ability to apply rules or schemas of word formation [24].

Despite a growing body of research showing evidence for a morphological disadvantage in dyslexia, studies on reading performance in poor readers have identified morphological abilities as a compensatory strategy for overcoming reading and spelling deficits [55, 56]. This line of research has proposed that the morpheme, as a unit of intermediate grain size, proves useful in processing all types of linguistic stimuli, including existing words, in individuals with limited reading skills (dyslexics and younger readers) who did not fully develop whole-word processing. Specifically, [56] found that dyslexic pupils, as well as younger children, exploit morphological cues not only in reading pseudowords but also in reading real words. They interpreted this finding as evidence that morphological parsing in reading can be even more useful for less skilled readers who have not mastered whole-word processing yet and cannot rely on a lexical (whole-word) reading unit.

The results obtained from languages with shallow and deep orthographies confirm that morphological parsing is a viable strategy for compensating for phonological deficits and difficulties to automatize reading. In light of these findings, it is extremely plausible that training in awareness of morphology could have a positive impact on reading and spelling, especially in individuals like dyslexics who struggle with the challenges posed by orthographic systems. Consistently, positive results have been reported in the seminal study by [5], who found that training in morphological awareness can aid reading-impaired adolescents to achieve better results in single-word and text reading, and spelling skills. Along the same lines are the results obtained by [57], finding that students with dyslexia who had received a morphological awareness treatment performed better than controls on morphological awareness tasks, reading comprehension, and spelling.

Other evidence in favor of a compensatory effect of morphological awareness skills in reading tasks is found in adults with dyslexia. Along the lines of [58, 59] found that dyslexic adults performed better than their reading-age matched controls and similarly to chronological-age matched controls in morphological tasks. However, they underperform their peers in phonological awareness tasks, suggesting that morphological awareness has a longer developmental trajectory than phonological awareness, and confirming the compensatory role of morphological skills in overcoming reading deficits. Consistently, [60] found a larger interaction between morphological awareness and word reading skills in adults with dyslexia when compared with typical readers, indicating the crucial role played by morphology in the reading-impaired population (see also the results of the meta-analyses by [31, 32]).

To conclude, a large body of evidence has shown that morphological awareness is impaired in children with dyslexia; nonetheless, deficits in this domain are more likely to be secondary to the major deficits in phonological awareness and processing characterizing dyslexia. Furthermore, studies on dyslexic adults have confirmed the different developmental trajectories of phonological and morphological awareness, with the latter being a late-emerging skill that continues to grow across school grades and turns out to play a compensatory role in overcoming reading impairments, at both initial and later stages of literacy development. In line with these findings, further research has emphasized the importance of morphological training, which could enhance reading abilities, being even more beneficial for poor readers and people with dyslexia.

### 1.3. A focus on Italian: Inflectional and derivational morphology

Our study stems from the line of investigation on morphological awareness of typically developing and dyslexic children outlined in the previous section and extends it to the acquisition of Italian morphology. The experimental design is inspired by Berko’s Wug test; hence, we made use of nonwords intending to test children’s morphological awareness skills while factoring out their vocabulary knowledge. However, different from English for which the Wug test was originally designed, Italian is a language with a rich array of inflectional and derivational morphemes, whose acquisition and full mastery are influenced by several variables such as (type) frequency, productivity, transparency, and regularity of word-formation processes. Since in our protocol we specifically assessed inflectional and derivational phenomena in the Italian nominal and verbal morphology, it can be useful to briefly illustrate its main characteristics.

Let’s consider inflection first. Being of fusional type, Italian morphology is characterized by suffixes that express sets of grammatical features (also called *portmanteau* morphemes). In the nominal (and adjectival) domain, we find declension classes, i.e., pairings of singular and plural endings having a more or less transparent relation with the gender and number feature they express.

A high degree of formal transparency is found when one ending is uniquely associated with a feature (or feature set) value: this is the case of the nominal suffix -*o*, which is almost invariably found in masculine singular nouns or of the nominal suffix -*a*, which is mostly found in feminine singular nouns (but there is a restricted class of nouns, mainly borrowings from Ancient Greek and Latin, which are masculine: e.g., *morfem-a* ‘morpheme’, *pirata* ‘pirate’). On the other hand, rather low transparency is found with the singular ending -*e*, which may be either masculine or feminine singular (*pont-e*_*MascSing*_ ‘bridge’ vs. *luc-e*_*FemSing*_ ‘light’), and a feminine plural marker (*port-e*_*FemPlu*_ ‘doors’). Formal transparency also correlates with higher frequency and productivity (i.e., use in new formations) of the corresponding declension classes, hence influencing the acquisition and processing of inflection (see [61]). Specifically, with declension class productivity, we refer to the ratio of the number/frequency of new formations of one Declension class and the total number/frequency of new formations (among the neologisms, we do not consider nominals formed via derivational affixes or compounding of existing forms).

Table 1 contains a schematic representation of the most common declension classes (to simplify the picture, invariables like *bar*, *koala*, lexical plurals like *uovo* / *uova* ‘egg/eggs’, and specificities of natural gender classes are excluded).

**Table 1:** Italian noun declension classes.

Decl. Class	Gender	Number	Ending	Example	Transl.	Transparency	Frequency	Productivity
**Cl. I**	Fem.	Sg.	-a	porta	door	high	high	high
		Pl.	-e	porte				
**Cl. II**	Masc.	Sg.	-o	giardino	garden	high	high	high
		Pl.	-i	giardini				
**Cl. III**	Masc. / Fem.	Sg.	-e	ponte_Masc_ / luce_Fem_	bridge / light	low	high	none
		Pl.	-i	ponti_Masc_ / luci_Fem_				
**Cl. IV**	Masc.	Sg.	-a	pirata	pirate	low	low	none
		Pl.	-i	pirati				

In our study, we tested both highly frequent, regular, and transparent declension classes (Class I and II) and non-transparent, unproductive classes, both frequent (Class III) and less numerically consistent (Class IV; for a more detailed discussion of the Italian morphological system and the declension classes we considered, see [14, 62–64]).

Again in the nominal domain, we tested the production of evaluative nouns eliciting the suffixes *-ino*, -*one*, and *-accio* (respectively diminutive, augmentative, and pejorative), as in *gattino* ‘little cat’, *gattone* ‘big cat’, *gattaccio* ‘nasty/ugly gat’, all derived from *gatto* ‘cat’. These word formation processes affect word semantics as it typically happens with derivation processes, even though lack of lexical category change or other feature values makes evaluative formation a process in between inflection and derivation proper.

In the current study, we especially focused on the verbal domain, where we assessed both inflectional and derivational phenomena. Acquiring Italian verbal morphology is especially challenging, since inflection endings are numerous, expressing a plurality of features (person, number, aspect/tense, and mood) and being different across conjugation classes. Parallel to nominal declension classes, verbal conjugations entail a high number of inflectional endings and correlate with a higher or lesser amount of root/stem allomorphy [65]. Indeed, conjugation classes exhibit different levels of frequency, productivity (referring to the ratio of the number/frequency of new verb formations of one conjugation class and the total number/frequency of new verb formations), and formal regularity, as shown in Table 2.

**Table 2:** Italian verb conjugation classes.

Conj. class	Theme Vowel	Example	Frequency	Productivity	Regularity
**Conj. I**	-a-	*amare* ‘to love’	high	high	high
**Conj. II**	-e-	*prendere* ‘to take’	mid-low	none	very low
**Conj. III**	-i-	*dormire* ‘to sleep’	mid-low	mid-low	mid-low

The examples in Table 2 are infinitives where conjugations are marked by three different theme vowels and -*re* is the infinitival morpheme (see [66]). Conj. I (-*are*, as in *amare* ‘to love’) is the most regular, frequent, and fully productive: most neologisms fall in this class, which is widely used with loanwords and most denominal verbs. Conj. III (-*ire*, *dormire* ‘to sleep’) is consistently less frequent and semi-productive, being especially employed with deadjectival verbs (*zittire* ‘to hush’, from *zitto* ‘silent’) but not with borrowings (*bluffare* ‘to bluff’, from *bluff*). Notice that the third conjugation class in -*ire* contains a subclass of verbs with infixed -*isc*-, as in *finire* / *fin-isc-o* ‘to end / I end’ (vs. *dormire* / *dorm-o* ‘to sleep / I sleep’): this subclass is more regular and stable than the other verbs in -*ire*. To the aims of the present study, however, the identification of this fourth subclass is irrelevant as no task directly taps the inflection features of this verbal paradigm. Conj. II (-*ere*, *prendere* ‘to take’) is also scarcely frequent, but it is totally unproductive and highly irregular, characterized by a high rate of root allomorphy, with only a few regular forms (see [62, 65, 67–69]).

The complicacies of conjugation classes also show up in derivation, with effects on the formal transparency and regularity of various derivation operations. To offer a concrete example here: derivational phenomena may entail the allomorphy of the base which, in the simplest case, manifests itself as a change in the stem-ending vowel in the derived forms. For instance, most verb stems do not change their form when used in category-changing derivations (e.g., *lavor-a-re* ‘to work’ > *lavora-tore* ‘worker’, *serv-i-re* ‘to serve’ > *serv-i-tore* ‘servant’); but those of the least regular conjugation class typically have their final vowel -*e* changed into -*i-* in derivation operations (e.g., *batt-e-re* ‘to hit, beat’ > *batt-i-tore* ‘batter’). Therefore, whereas verb-based derivation usually preserves the theme vowel of the verb of Conj. I and III (-*a*- and -*i*-), Conj. II emerges as the least transparent, with -*e*- being systematically lost in many derivational phenomena: *scorr-e-re* ‘to flow’> *scorr-i-mento* ‘flow’, *legg-e-re* ‘to read’ > *legg-i-bile* ‘readable’, etc. Some other phenomena are more idiosyncratic, as in the case of *legg-e-re* > *let-tore* ‘reader’, where the verbal stem of the infinitive changes more radically and unpredictably in the nominalization process (in our experiment, dealing with nonwords, we will stick to the ‘regular’ cases of inflection and derivation, replicating the most common and predictable patterns in Italian morphology).

Therefore, considering transparency, frequency, productivity, and regularity across types of morphological phenomena is especially intriguing in a language like Italian where declension classes in the nominal system and conjugation classes in the verbal system vary along these dimensions. In light of the acquisitional and processing challenges associated with these aspects of Italian morphology, we believe this language is a particularly interesting testing ground for measuring the morphological awareness of children with both typical and atypical development.

### 1.4. Research aims and predictions

Our experimental protocol aimed to provide a comprehensive assessment of Italian typically developing and dyslexic children’s morphological skills. To achieve this main goal, we measured various types of morphological processes and variables, and assessed their (potential) degree of disruption in dyslexia. Specifically, our study addressed three research aims.

The **first aim** was to compare the morphological awareness abilities of children with developmental dyslexia with those of their typically developing peers. Based on the results reported in the literature and reviewed above, we expected children with dyslexia to display more marked difficulties than their peers across different types of tasks.

Moreover, in our **second aim**, we were especially interested in comparing the nominal and verbal domains: while on the former there has been previous research [14, 64], the latter is relatively less explored and, thus far, no studies on verb-based phenomena have involved Italian children with dyslexia. Our expectations, however, were that tasks implying the manipulation of verbs and verb-based forms would be more taxing across groups, compared to those involving nouns, especially due to the high morphological complexity of Italian verb-based word formation (see section 1.3).

The **third aim** was to assess morphological awareness within a multidimensional approach encompassing and scrutinizing two main aspects, defined as follows: 1) **type of process**, i.e., comparing inflection vs. derivation, the former being by far the most investigated domain up until now with less attention devoted to the latter; 2) ‘**directionality’ in the operation**, i.e., comparing the production of a complex form from a base word with the retrieval of a simpler or more basic form from a more complex one, to understand if there are differences between the two types of operations. The reason for addressing these two aspects, until now underinvestigated, was to verify whether children manifest more marked difficulties in one domain over the other and what the differences, if any, are between typical and atypical children. Our predictions about these finer-grained aspects of morphological competence were less straightforward. While more is known about inflection, derivation is comparatively less explored, especially in Italian; moreover, to the best of our knowledge, there is no specific work comparing the role played by the directionality of the process, i.e., whether it is simpler to identify the infinitival form of a verb-based complex form or the other way around (which makes sense under the assumption that words are created dynamically, combining morphemes along rules or schemata of word formation). Since, compared to inflection, derivation is more irregular, less frequent, and acquired later in typical development, we expected to find derivational tasks rather challenging and, possibly, more challenging than inflectional tasks for all children. Moreover, we expected tasks requiring sophisticated skills, like production and base retrieval of nonwords (entailing a conscious reflection upon word structure) from irregular and infrequent conjugation classes to be difficult for all children, and especially for children with dyslexia.

Finally, the **fourth aim** was to identify and measure the potential effects of transparency, frequency, and productivity across different types of morphological phenomena, in children with and without dyslexia. To this aim, we manipulated conditions based on conjugation classes, which, as explained in the introduction, manifest different degrees of transparency, frequency, and productivity in Italian morphology. Since we have already extensively investigated the differences across declension classes in the nominal domain in our previous works [14, 64, 70], in this study we were particularly interested in exploring the verbal domain and its conjugation classes. In this respect, we expected all children to have worse performances with less transparent operations and, specifically, less frequent/productive conjugation classes, not only in inflection but also in derivation (in both production and base retrieval). However, because of the deficits of children with dyslexia reviewed in section 1.2, we expected reading-impaired children to underperform their typically developing peers, particularly with the word forms whose production requires more sophisticated skills. Specifically, based on the peculiarities characterizing the Italian nominal and verbal morphology presented in section 1.3., we expected difficulties to be higher in both the inflection and the derivation of nonwords belonging to infrequent, unproductive, and irregular classes. As for the verbal domain, besides frequency factors which could favor Conjugation I in *-are* over the less common and regular conjugations in *-ire* and, especially, *-ere*, difficulties were expected to be particularly marked where there is a low degree of formal transparency because a vowel change is required, as in past participle formation from Conjugation II (e.g., *tem-e-re* ‘to fear’ > *tem-u-to* ‘feared’). Although we expected lower accuracy rates with the least transparent, frequent, and productive classes from all children, comparing typical and dyslexic children’s performance across the different tasks can provide interesting data about how dyslexia interacts with different degrees of transparency, frequency, and productivity of morphological operations.

To accomplish these research aims, we designed a comprehensive experimental protocol, as will be illustrated more in detail below, which we administered to dyslexic and typically developing age-matched children, including different tasks that required the manipulation of nonwords, allowing us to tackle morphological processes while filtering out the effects of lexical knowledge. The advantage of testing a morphologically rich language like Italian, displaying various inflectional and derivational morphemes, is that it can provide a more fine-tuned investigation of children’s morphological awareness, whose contribution to the development of reading abilities is critical especially for the reading-impaired profiles.

## 2. Methods

### 2.1. Participants

The experimental protocol was administered to 45 subjects, divided into two groups: 21 dyslexic children (mean age 9;10 years old, SD = 1.3) and 24 age-matched typically developing control children (mean age 10;3, SD = 0.83). An independent sample t-test revealed that there were no significant differences in the age of the subjects (*t*(34.652) = 1.352, *p* = .185).

All children attended the same public schools in Northeast Italy and were monolingual speakers of Italian. Children with dyslexia had been independently diagnosed as dyslexic on standard criteria (ICD-10; World Health Organization, 2004) and they had no diagnosed or reported oral language problems, suggesting that they were not suffering from possible comorbidity with SLI/DLD (Specific Language Disorder/Developmental Language Disorder). Typically developing children had no diagnosed or referred cognitive deficit, no language problems, hearing disorders, or reading difficulties. All children, both dyslexics and controls, had normal or corrected to normal vision. The study was approved by the local ethics committee and conducted in accordance with the standards specified in the 2013 Declaration of Helsinki; written informed consent was given by the parents of all the children who participated in the study.

To assure comparability of the groups, all subjects were administered a set of preliminary tasks including the CPM Raven, assessing nonverbal intelligence (no subject scored below 1.5 SD under the mean for their age, following the Italian standardization provided by [71]), and the *Peabody Picture Vocabulary Test* (PPVT-R, Italian standardization by [72]), assessing their receptive vocabulary (no subject scored below 1.5 SD under the mean for their age). We also assessed the participants’ reading abilities, by administering Tasks 2 and 3 of the DDE-2 [73], a standardized task that assesses speed and accuracy in word and nonword reading. As an inclusion criterion, dyslexic children had to score below 2 SD under the mean of their age in at least two of the four measures administered (accuracy and speed of word and nonword reading of the DDE-2), whereas none of the typically developing children had to score below 1.5 SD in any of the tasks administered.

The results of the preliminary measures of the two groups are summarized in Table 3.

**Table 3:** Means (and SDs) of the two groups in nonverbal intelligence (CPM Raven), vocabulary (PPVT-R), and word and nonword reading (DDE-2).

Group	Nonverbal Intelligence (z-scores)	Vocabulary (raw scores)	Word Reading Accuracy (z-scores)	Word Reading Speed (z-scores)	Nonword Reading Accuracy (z-scores)	Nonword Reading Speed (z-scores)
**Dys**	0.68	108.62	-2.58	-4.05	-2.12	-2.53
	(0.73)	(11.65)	(1.85)	(3.04)	(1.48)	(2.12)
**Con**	0.41	111.17	0.45	0.64	0.41	0.41
	(0.81)	(13.47)	(0.74)	(0.65)	(0.70)	(0.70)

Note. Dys = Dyslexic children; Con = Control children

A series of independent sample t-tests revealed that there were no significant differences in the CPM Raven task (*t*(43) = 1.206, *p* = .234) and in the PPVT-R (*t*(32) = .674, *p* = .504). The good performance of the dyslexic group in the vocabulary task, together with the absence of language disorders reported by the health professionals who ran the diagnosis (neuropsychiatrists, psychologists, and speech therapists) as well as by teachers and parents, allows us to exclude the presence of comorbidities with SLI/DLD. Conversely, significant group differences were found in word reading speed (*t*(21.599) = 7.023, *p* < .001), word reading accuracy (*t*(25.645) = 7.023, *p* < .001), nonword reading speed (*t*(26.030) = 5.519, *p* < .001) and nonword reading accuracy (*t*(27.677) = 7.149, *p* < .001), confirming the presence of severe reading deficits in the children with dyslexia.

The presence of a control group composed of children of the same age, intelligence level, and vocabulary skills as the participants with dyslexia allows us to affirm that possible differences in the morphological tasks between the two groups are not due to differences in age, intelligence, and vocabulary but they are rather related to the presence of a specific reading disorder.

### 2.2. Materials

To provide an in-depth assessment of the children’s morphological competence, we developed 11 tasks requiring the manipulation of nonwords and addressing different types of morphological processes (inflection vs. derivation), with different directionality of the operation (production vs. base retrieval) and requiring manipulation within the nominal or the verbal domain respectively by means of pseudo-nouns or pseudo-verbs, as summarized in Table 4.

**Table 4:** Characteristics of the 11 tasks: Type of process, directionality, lexical domain involved.

Task	Process Type	Process Directionality	Lexical domain involved
**Task 1: Nonword pluralization**	Inflection	Production	Nominal
**Task 2: Past-Participle Inflection**	Inflection	Production	Verbal
**Task 3: Deverbal nouns in -*tore***	Derivation	Production	Verbal
**Task 4: Deverbal nouns in *-mento***	Derivation	Production	Verbal
**Task 5: Deverbal nouns in -*ta***	Derivation	Production	Verbal
**Task 6: Deverbal adjectives in *-bile***	Derivation	Production	Verbal
**Task 7: Evaluative Nouns**	Derivation	Production	Nominal
**Task 8: Verb base retrieval from past participle**	Inflection	Base retrieval	Verbal
**Task 9: Verb base retrieval from nouns in *-tore***	Derivation	Base retrieval	Verbal
**Task 10: Verb base retrieval from nouns in *-ta***	Derivation	Base retrieval	Verbal
**Task 11: Verb base retrieval from adjectives in *-bile***	Derivation	Base retrieval	Verbal

**Fig 1:**
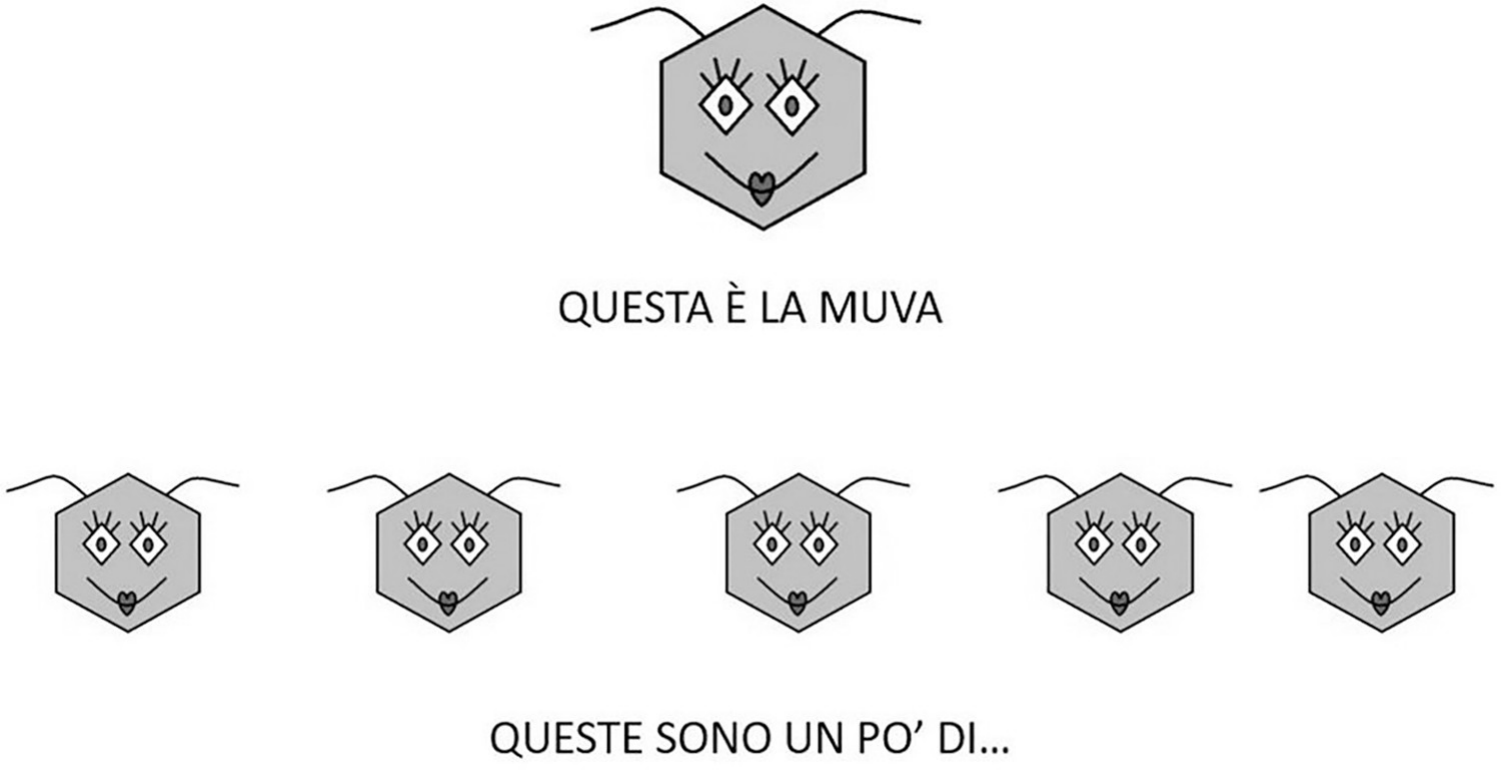
Representation of an experimental item administered in Task 1 addressing the pluralization of invented nouns.

The tasks were administered as follows: each child was explained that she was going to participate in a game involving invented words, that she was about to listen to some short stories with weird characters or actions, and that she simply had to complete some sentences playing with these invented words. All the experimental stimuli were presented on a laptop computer; the child was shown some pictures and heard a pre-recorded voice of a feminine native speaker of Italian presenting each of the stimuli and uttering an incomplete statement which she was instructed to complete, as in the example reported below, where the participant hears the elicitation formula in (1) while looking at the picture reported in Fig 1.

1. Questa è la muva. Queste sono un po’ di… (target: *muve*)

‘This is *la muva*. These are some… (target: *muve*)”

In the case of invented nouns, the picture portrayed an invented character (as in Fig 1), while for invented verbs the picture simply portrayed the subject of the sentence (e.g., a girl). As shown in Fig 1, below each picture the relevant nonword was presented on the screen also in its written form. This was intended to aid memorization and avoid the burden of correctly remembering the nonword.

Overall, 121 nonwords were deployed; they were carefully designed in compliance with the phonotactic rules of Italian and manually created by combining high and low-frequency type syllables taken from an Italian syllable frequency database [74]. The orthographic similarity of the test items was measured using the NIM web-based software (see [75] for the technical details). The mean value of orthographic similarity of all the test items designed for our protocol was 0.25 (SD = 0.16); notice that in some cases the similarity was increased by the presence of the same suffix, as in *famp-a-****tore*** and *fol-i-****tore***, which has a similarity of 0.70). To exclude possible confounding effects, within each task the similarity of the test items was always lower than 0.75 (the maximum similarity score was 0.72 in Task 3 between *taspere* and *talmire*).

Our protocol included 11 tasks assessing the nominal (with 2 tasks) and especially the (de-)verbal domains (i.e., regarding lexical items built from, or deriving, verbs, with 9 tasks), with conditions designed on the dimensions of frequency, productivity, regularity, and formal transparency of the word formation phenomena considered, along the lines illustrated in section 1.3 above.

Concerning the nominal domain, we tested the pluralization of nonwords belonging to all four declension classes in Table 1; Class III was split into two conditions based on the gender value of the nonword (e.g., *il dorte*_*Masc*_, *la stabe*_*Fem*_), yielding a total of 5 conditions, as in [14]. As for the derivation of nouns from nominal bases, we tested the production of evaluative nouns eliciting the suffixes *-ino*, -*one*, and *-accio* (respectively diminutive, augmentative, and pejorative). Finally, as for the (de-)verbal domain, we assessed both inflectional and derivational phenomena, considering all three Italian conjugation classes; we also tested the children’s ability in base-retrieval tasks requiring them to identify the target verb form from a complex pseudoword.

We will now describe each of the 11 tasks administered; for convenience, a summary of the elicitation formulas and experimental conditions, with an example of each condition is reported in Table 5.

**Table 5:** The tasks: Elicitation formula, conditions, and examples.

TASK	ELICITATION FORMULA	CONDITIONS	EXAMPLES
**TASK 1** **Nonword pluralization**	*Questa è* (*la* muva), *queste sono un po’ di …(target*: muve*)* (‘This is la muva_FemSing_, these are some…muve_FemPlur_’)	1. N -*a*_FemSing_>-*e*_FemPlu_ (Class I; 3 items) 2. N -o_MascSing_>-i_MascPlu_ (Class II; 3 items) 3. N -*a*_MascSing_>-*i*_MascPlu_ (Class IV; 3 items) 4. N -e_MascSing_>-*i*_MascPlu_ (Class III/Masc; 3 items) 5. N -e_FemSing_>-*i*_FemPlu_ (Class III/Fem; 3 items)	1. la muv-a > muv-e 2. il fol-o > fol-i 3. il tred-a > tred-i 4. il dort-e > dort-i 5. la stab-e > stab-i
**TASK 2** **Past-participle inflection**	*Qui si è messo a pindare*. *Cos’ha fatto*?” (target: *Ha pind-ato*). (‘Here he started to *pindare*_Inf_. What has he done? He has… *pindato*_PastPart_)	1. V -are_Inf_> -ato_PastPart_ (Conj. I; 3 items) 2. V -ere_Inf_> -uto_PastPart_ (Conj. II; 3 items) 3. V ire_Inf_> -ito_PastPart_ (Conj. III; 3 items)	1. pind-are > pind-ato 2. nov-ere > nov-uto 3. call-ire > call-ito
**TASK 3** **Deverbal nouns in -*tore***	“*Questa persona ama molto faspare*, *quindi possiamo dire che è un… (target*: *faspatore)”* (‘This person really loves *faspare*_Inf_, then we can say that he is a…*faspatore*_Noun’_).	1. V -*are*_Inf_ > -atore_Noun_ (Conj. I; 3 items) 2. V -*ere*_Inf_ > -itore_Noun_ (Conj. II; 3 items) 3. V -*ire*_Inf_ > -itore_Noun_ (Conj. III; 3 items)	1. fasp-a-re > fasp-a-tore 2. mop-e-re > mop-i-tore 3. pon-i-re > pon-i-tore
**TASK 4** **Deverbal nouns in -*mento***	“*Questa bambina vuole sempre budare*, *dopo un po’ vedremo il suo*… (expected: budamento)” (‘This girl always want to *budare*_Inf_, in a while we will see her…*budamento*_Noun_’).	1. V -*are*_Inf_ >*a-mento*_Noun_ (Conj. I; 3 items) 2. V -*ere*_Inf_ >*i-mento*_Noun_ (Conj. II; 3 items) 3. V -*ire*_Inf_ >*i-mento*_Noun_ (Conj. III; 3 items)	1. bud-a-re > bud-a-mento 2. mal-e-re > mal-i-mento 3. vud-i-re > vud-i-mento
**TASK 5** **Deverbal nouns in *-ta***	“*A questa persona piace molto ponare*, *ieri si è fatta una bella…*(target: *ponata*)” (‘This person really likes *ponare*_Inf_, yesterday he had a nice…*ponata*_Noun_’)	1. V -*are*_Inf_ > -*a-ta*_Noun_ (Conj. I; 3 items) 2. V -*ere*_Inf_ > -*u-ta*_Noun_ (Conj. II; 3 items) 3. V -*ire*_Inf_ > -*i-ta*_Noun_ (Conj. III; 3 items)	1. pon-a-re > pon-a-ta 2. manf-e-re > manf-u-ta 3. fun-i-re > fun-i-ta
**TASK 6** **Deverbal adjectives in -*bile***	*Questa strada si può madare*, *quindi possiamo dire che è* (target: *madabile*)” (‘This street can be *madare*_*Inf*_, then we can say that it is…*madabile*_Adj_’)	1. V -*are*_Inf_ *>a-bile*_Adj_ (Conj. I; 3 items) 2. V -*ere*_Inf_ *>i-bile*_Adj_ (Conj. II; 3 items) 3. V -*ire*_Inf_ *>i-bile*_Adj_ (Conj. III; 3 items)	1. mad-a-re>mad-a-bile 2. lesp-e-re>lesp-i-bile 3. vasch-i-re>vasch-i-bile
**TASK 7** **Evaluative nouns**	*Questo è un fespo*. *Se è piccolo e magro possiamo dire che è un…*(target: fesp-ino_Dim_). S*e è grande e grosso possiamo dire che è un* (target: *fesp-one*). *Se è brutto e cattivo possiamo dire che è un…*(target: *fesp-accio*)” (‘This is a *fespo*. If it is small and tiny, we can say that it is a… (fespino_Dim_). If it is big and fat, we can say that it is a… (*fespone*_Aug)_. If it is ugly and evil, we can say that is a… (fespaccio_Pej_)’	1. N -*o*_MascSing_> -*ino*_Dim_ (3 items) 2. N -*o*_MascSing_ > -*one*_Aug_ (3 items) 3. N -*o*_MascSing_ > -*accio*_Pej_ (3 items)	1. gab-o > gab -ino_Dim_ 2. gab-o > gab -one_Aug_ 3. gab-o > gab -accio_Pej_
**TASK 8** **Verb base retrieval from past participles**	“*Questa bambina ha rimbato perché aveva voglia di…* (target: *rimbare*)” (‘This girl has rimbato_PastPart_, because she wanted to… *rimbare*_Inf_’).	1. V -*ato*_PastPart_> -*are*_Inf_ (Conj. I; 3 items) 2. V -*uto*_PastPart_> -*ere*_Inf_ (Conj. II; 3 items) 3. V -*ito*_PastPart_> -*ire*_Inf_ (Conj. III; 3 items)	1. rimb-a-to > rimb-a-re 2. panf-u-to > panf-e-re 3. sord-i-to > sord-i-re
**TASK 9** **Verb base retrieval from nouns in -*tore***	“*Al pifatore piace*…(target: *pifare*)” (‘The *pifatore* likes… *pifare*_Inf_’)	1. N -*a-tore*_Noun_*>a-re* (Conj. I; 3 items) 2. N -*i-tore*_PastPart_*>e-re/i-re* (Conj. II and III; 3 items)	1. pif-a-tore > pif-a-re 2. perg-i-tore>perg-e-re/perg-i-re
**TASK 10** **Verb base retrieval from nouns in -*ta***	“*Questo bambino si è fatto una bella vordata perché gli piace molto…*(target: *vordare*)” (‘This boy has done a nice *vordata*_PastPart_, since he likes…vordare_Inf_)	1. N -ata>are_Inf_ (Conj. I; 3 items) 2. N -uta>ere_Inf_ (Conj. II; 3 items) 3. N -ita>ire_Inf_ (Conj. III; 3 items)	1. vord-a-ta > vord-a-re 2. ram-u-ta > ram-e-re 3. fosp-i-ta > fosp-i-re
**TASK 11** **Verb base retrieval from adjectives in -*bile***	“*Questo foglio è garnibile perché si può…*(target: *garnire* or *garnere*)”. (‘This sheet is *garnibile*, because you can…(*garnire/ garnere*_Inf_) it’).	1. Adj -*a-bile*>are_Inf_ (Conj. I; 3 items) 2. Adj -*i-bile*>ire_Inf_ (Conj. II and III; 3 items)	1. mit-a-bile > mit-a-re 2. garn-i-bile > garn-e-re/ garn-i-re

#### Task 1, noun pluralization

This task was the same administered by [70] with monolingual and bilingual children, in turn, adapted from [14]. Subjects were presented with a fictitious character with an invented noun (e.g., *la gora*) and were asked to produce the plural (*le gore*). As shown in Table 5, five conditions were assessed.

#### Task 2, past participle inflection

This task required the subject to derive the past participle of a nonce verb. The child was presented with a character, Goofy, who performed some invented actions. As can be noted in Table 4, a vowel change is required in Conj. II, whereas the theme vowel is maintained in Conj. I and III.

#### Task 3, derivation of deverbal nouns in -tore

In this task, the subject was asked to derive an agentive noun from the infinitive form of a nonce verb, by adding the suffix *-tore* to the base form of the verb (as *pescatore* ‘fisher’ from *pescare* ‘to fish’). To produce the correct agentive nouns, the theme vowel of the verb must be produced together with the suffix -*tore* for Conj. I and III, whereas in Conj. II there is a vowel change (*e* > *i*, as in *vinc-****i****-tore* ‘winner’ from *vinc-****e****-re* ‘to win’).

#### Task 4, derivation of deverbal nouns in -mento

This task required the subject to derive, from the infinitive form of the verb, the form of the corresponding action nominal by adding the suffix *-mento*. In this case, too, deriving a noun from a verb in Conj. II requires a change in the theme vowel (*e* > *i*, as in *combatt-****i****-mento* ‘fight’ from *combatt-****e****-re* ‘to fight’).

#### Task 5, derivation of deverbal nouns in -ta

This task requires the subject to derive, from the infinitive form of the verb, the corresponding form of the eventive nominal (i.e., a deverbal noun denoting the single event expressed by the verb), by adding the suffix *-ta* to the base form of the verb. Also in this case there is a vowel change in Conj. II (*e* > *u*, as in *cad-****u****-ta* ‘fall’ from *cad-****e****-re* ‘to fall’).

#### Task 6, derivation of deverbal adjectives in -bile

In this task, the subject is asked to derive an adjective from the infinitive form of a nonce verb by adding the suffix *-bile*. Again, a vowel change is required for verbs of Conj. II, which take the same vowel as verbs of Conj. III (as in *otten-****i****-bile* ‘gettable’ from *otten-****e****-re* ‘to get’).

#### Task 7, derivation of evaluative nouns

This task required the subject to derive from the base form of the noun the corresponding diminutive, augmentative and pejorative forms of the noun itself, by adding respectively the suffixes *-ino*, *-one*, and *-accio*, which are typically used in Italian morphology to express dimensional and/or evaluative properties of the entity referred by the noun. For homogeneity, we used only masculine invented nouns ending in -*o*.

#### Task 8, verb base retrieval from past participles

This is a task of inflectional morphology in which the subject is required to produce the corresponding infinitive form of the verb from the past tense of nonce verbs.

#### Task 9, verb base retrieval from nouns in -tore

This task required the subject to produce, from the nouns ending in *-tore*, the infinitive form of the corresponding verbs, by eliding the suffix -*tore* and adding the infinitive ending *-re* to the verb stem (hence by selecting the correct theme vowel). In this case, we had 6 items, 3 for each of the two conditions corresponding to Conj. I and III, since only derived forms in *-a-tore* and *-i-tore* are allowed in Italian. However, given that nouns ending in *-i-tore* may have been derived from a verb ending in *-ere* (e.g., *vinc-****e****-re* ‘to win’ from *vinc-****i****-tore* ‘winner’), we considered correct also answers containing a verb belonging to Conj. II.

#### Task 10, verb base retrieval from nouns in -ta

In this task, the subject had to produce an infinitive from deverbal nouns ending in *-ta*. To do so, the child has to identify the conjugation class of the verb, elide the suffix *-ta*, and add the typical infinitive ending -*re* preceded by the correct theme vowel; notice that also in this case there is a vowel change for items belonging to Conj. II (e.g. *batt-****u****-ta* ‘beating/serve’, from *batt-****e****-re* ‘to beat/serve’).

#### Task 11, verb base retrieval from adjectives in -bile

In this task, the child had to retrieve the infinitive from a given invented adjective in *-bile*, by eliding the suffix and adding the infinitive in -*re* preceded by the correct theme vowel. Only adjectives in *-a-bile* and *-i-bile* are allowed in Italian, with verbs of Conj. II and III yielding *-i-bile*. Therefore, for -*i-bile* adjectives, we considered correct also answers containing a verb belonging to Conj. II.

### 2.3. Procedure and scoring system

Each child was individually tested in a quiet room and was instructed to answer by uttering the relevant inflected, derived, or infinitive item through a short training provided for each task first with words and then with nonwords. During this familiarization phase, children were given feedback to make sure that they understood the task. No feedback was given during the experimental phase. The tasks of the whole protocol, including the preliminary measures presented in section 2.1, were administered in the following order: nonverbal intelligence test (CPM Raven), morphological tasks 3-4-5-6, reading tasks (DDE-2), morphological tasks 1-2-7-8-9, receptive vocabulary (PPVT-R), morphological tasks 10–11. The same order was used in each administration. Each task comprised three items per condition and two or three training items, depending on the task. The different conditions and trials within each task were presented in randomized order. The whole experimental session took around 45 minutes.

As for the scoring system of the morphological tasks, following [14, 70], 1 point was awarded for each correct item and 0 points for incorrect ones; no penalties were given for mispronunciation errors if the target morphological operation was correctly performed (e.g., il tre**d**a > i tre**b**i). This was intended to avoid penalization effects, especially for children with dyslexia who might display difficulties in correctly repeating nonwords due to their phonological impairments, as discussed in section 1.2.

All tests were coded twice by both authors; the few disagreements in the coding were resolved after a discussion between the coders. The interrater reliability was 97% (Cronbach’s alpha = 0.97).

## 3. Results

### 3.1. Data analysis plan

Four different analyses were run on the data collected to address the research aims outlined in 1.4., using the statistical environment R [76] and in particular the packages lme4 and lmerTest [77, 78]. In **Analysis 1**, to compare the morphological awareness abilities of children with developmental dyslexia with those of their typically developing peers in the different tasks, we calculated a series of generalized linear mixed effects regression models with the overall accuracy in each of the 11 tasks as dependent variable and Group (dyslexics vs. controls) as fixed effect, adding Participant and Item as crossed random effects.

In **Analysis 2**, intending to compare the verbal and the nominal domains, we ran a generalized linear mixed effects regression model with accuracy as dependent variable, Group (dyslexics vs. controls) and Category (nouns vs. verbs) as fixed effects, and Participant and Item as crossed random effects. In Analyses 3 and 4, instead, we aimed at providing a more fine-grained analysis of morphological processes in the verbal domain, while also addressing the role of the three conjugation classes (-*are*, -*ere*, and -*ire*). For both analyses, we only considered Tasks 2, 3, 4, 5, 6, 8, and 10: Tasks 1 and 7 were excluded as they addressed the nominal domain, while Tasks 9 and 11 were excluded since, being base retrieval tasks, they allowed for answers containing verbs belonging to both Conj. II and III. More particularly, in **Analysis 3**, we compared the performance of the two groups in the three conjugation classes across the two morphological processes (inflection vs. derivation; respectively: Tasks 2 and 8 for inflection and Tasks 3, 4, 5, 6, 10 for derivation); to do so, we ran a generalized linear mixed regression model with accuracy as dependent variable, adding Group (dyslexics vs. controls), Condition (-*are*, -*ere*, -*ire*) and Type of Process (Inflection vs. Derivation) as fixed effects and Participant and Item as crossed random effects. Posthoc tests were conducted using the emmeans() function in R with Tukey correction [79].

Finally, in **Analysis 4**, we aimed at analyzing performance in the three conjugation classes, comparing tasks requiring the production of inflected and derived forms (Tasks 2, 3, 4, 5, and 6) and tasks requiring base retrieval from inflected and derived words (Tasks 8 and 10). We then ran a generalized linear mixed regression model with accuracy as dependent variable, Group (dyslexics vs. controls), Condition (-*are*, -*ere*, -*ire*), and Directionality of Operation (Production vs. Base retrieval) as fixed effects and Participant and Item as crossed random effects.

### 3.2. Analysis 1

As shown by the data reported in Table 6 and summarizing the two groups’ performance in the 11 Tasks, children with dyslexia were overall less accurate than controls in inflecting and deriving nonwords. Interestingly, difficulties seem to be lower in the nominal domain for both groups, as shown by their higher performance in Task 1 (noun pluralization) and Task 7 (derivation of evaluative nouns). Conversely, the gap between dyslexics and controls seems to be higher in derivational tasks involving base retrieval.

**Table 6:** General mean accuracy (SDs) in all morphological tasks.

Group	Task 1	Task 2	Task 3	Task 4	Task 5	Task 6	Task 7	Task 8	Task 9	Task 10	Task 11
**Con**	0.82 (0.38)	0.64 (0.48)	0.71 (0.46)	0.62 (0.49)	0.51 (0.50)	0.63 (0.48)	0.93 (0.26)	0.70 (0.46)	0.84 (0.37)	0.69 (0.46)	0.86 (0.35)
**Dys**	0.70 (0.46)	0.46 (0.50)	0.58 (0.50)	0.51 (0.50)	0.37 (0.48)	0.50 (0.50)	0.83 (0.38)	0.58 (0.50)	0.55 (0.50)	0.52 (0.50)	0.67 (0.47)

*Note*: Con = Control children; Dys = Dyslexic children. Task 1 = Nonword pluralization; Task 2 = Past-participle inflection; Task 3 = Deverbal nouns in -*tore*; Task 4 = Deverbal nouns in -*mento*; Task 5 = Deverbal nouns in -*ta*; Task 6 = Deverbal adjectives in -*bile*; Task 7 = Evaluative nouns; Task 8 = Verb base retrieval from past participles; Task 9 = Verb base retrieval from nouns in -*tore*; Task 10 = Verb base retrieval from nouns in -*ta*; Task 11 = Verb base retrieval from adjectives in -*bile*.

The best fitting models showed the presence of a significant main effect of Group, with dyslexics being less accurate than controls in Task 1 (noun pluralization; β = -1.20, SE = 0.52, z = -2.31, p < .05), Task 2 (past participle inflection; β = -0.94 SE = 0.31, z = -3.06, p < .01), Task 3 (derivation of deverbal nouns in -*tore*, β = -0.95, SE = 0.46, z = -2.06, p < .05), Task 5 (derivation of deverbal nouns in *-ta*, β = -0.87, SE = 0.30, z = -2.90, p < .01), Task 8 (verb base retrieval from past participle, β = -1.30, SE = 0.46, z = -2.86, p < .01), Task 9 (verb base retrieval from nouns in *-tore*, β = -1.76, SE = 0.42, z = -4.23, p < .001), Task 10 (verb base retrieval from nouns in -*ta*, β = -1.58, SE = 0.51, z = -3.11, p < .001) and Task 11 (verb base retrieval from adjectives in *-bile*, β = -1.66, SE = 0.62, z = -2.67, p < .01). The difference between dyslexics and controls instead approaches significance in Task 4 (derivation of deverbal nouns in -*mento*, β = -0.63, SE = 0.37, z = -1.70, p = .089), Task 6 (derivation of deverbal adjectives in *-bile*, β = -0.67, SE = 0.36, z = -1.86, p = .062) and Task 7 (derivation of evaluative nouns, β = -1.02, SE = 0.60, z = -1.71, p = .086). To summarize, the results of Analysis 1 indicated that dyslexics were generally less accurate than control children in the tasks administered, thus confirming the presence of extensive morphological deficits in dyslexia.

### 3.3. Analysis 2

In Analysis 2, we aimed at comparing the two groups in the two lexical categories addressed in our study, namely, nouns and verbs (considering under these labels all tasks that are based on pseudo-nouns and tasks that are based on pseudo-verbs). As shown in Table 7, both groups were more accurate with nouns than with verbs, and dyslexics were overall less accurate.

**Table 7:** Mean accuracy (SDs) of the two groups in nouns and verbs.

Group	Nouns	Verbs
**Con**	0.87 (0.34)	0.68 (0.47)
**Dys**	0.75 (0.44)	0.52 (0.50)

*Notes*. Con = Control Children; Dys = Dyslexic Children. The tasks considered are Tasks 1 and 7 for morphological operations involving nouns and Tasks 2, 3, 4, 5, 6, 8, 9, 10, 11 for those involving verbs.

We calculated a generalized linear mixed regression model with accuracy as dependent variable, adding Group (dyslexics vs. controls) and Category (Nouns vs. Verbs) as fixed effects and we added Participant and Item as random intercepts. The type of process (Inflection vs. Derivation) as well as the interaction between Group and Category were not included as they did not contribute to the model’s fit. The best fitting model showed a significant main effect of Group, with dyslexics being less accurate than controls (β = -0.95, SE = 0.19, z = -4.97, p < .0001) and a main effect of Category, with accuracy being lower with verbs than with nouns (β = -1.39, SE = 0.34, z = -4.13, p < .0001).

In summary, both groups of children had more difficulties in inflecting and deriving verbs compared to nouns, and dyslexics were generally less accurate than controls.

### 3.4. Analysis 3

In Analysis 3, we aimed at comparing groups in derivational and inflectional tasks, while also considering the role of the three conjugation classes. As shown in Table 8, the performance of both groups was generally higher in inflection than derivation, with difficulties being particularly marked with verbs in -*ere*; moreover, dyslexics were generally less accurate than controls.

**Table 8:** Mean accuracy (SDs) of the two groups in inflectional and derivational tasks in the three conjugation classes.

Group	Inflection (total)	Derivation (total)	Inflection -*are*	Inflection -*ere*	Inflection -*ire*	Derivation -*are*	Derivation -*ere*	Derivation -*ire*
**Con**	0.67 (0.47)	0.63 (0.48)	0.90 (0.31)	0.33 (0.47)	0.78 (0.42)	0.78 (0.42)	0.32 (0.47)	0.81 (0.40)
**Dys**	0.52 (0.50)	0.50 (0.50)	0.83 (0.37)	0.12 (0.33)	0.60 (0.49)	0.67 (0.47)	0.23 (0.42)	0.59 (0.49)

*Notes*. Con = Control Children; Dys = Dyslexic Children. We considered Tasks 2 and 8 for inflection and Tasks 3, 4, 5, 6, 10 for derivation)

To evaluate the performance of the two groups comparing the two morphological processes, while also addressing the role of the three conjugation classes in accuracy rates, we ran a generalized linear mixed regression model with accuracy as dependent variable, adding Group (dyslexics vs. controls), Conjugation (-*are*, -*ere*, -*ire*) and Type of Process (Inflection vs. Derivation) as fixed effects with full interactions and Participant and Item as crossed random factors.

We found a significant main effect of Group, with dyslexics being less accurate than controls (β = -0.64, SE = 0.26, z = -2.50, p < .05), a main effect of Type of Process, with accuracy being higher in inflectional than in derivational tasks (β = 0.99, SE = 0.46, z = 2.17, p < .05) and a main effect of Conjugation (χ^2^ = 84.38, df = 2, p < .0001); the interaction between Group and Conjugation was also significant (χ^2^ = 7.69, df = 2, p < .05). The remaining interactions were not significant: Group*Type of Process (χ^2^ = 0.01, df = 1, p = .978), Type of Process*Conjugation (χ^2^ = 3.82, df = 2, p = 0.15), and Group*Type of Process*Conjugation (χ^2^ = 5.32, df = 2, p = .07).

To investigate the nature of the Group*Conjugation interaction, data were split according to Group and the effect of Conjugation was investigated in these subsets. Results of the posthoc tests showed that for dyslexics difficulties were higher with verbs in -*ere* than with verbs in -*are* (ß = -2.87, SE = 0.33, z = 8.73, p < .0001) and -*ire* (ß = -2.15, SE = 0.32, z = -6.74, p < .0001) and that verbs in -*ire* were more difficult than verbs in -*are*, although the difference was only marginally significant (ß = -0.72, SE = 0.31, z = -2.34, p = .051). As for controls, instead, differences were found between -*ere* and -*are* (ß = -2.53, SE = 0.24, z = -10.41, p < .0001), -*ere* and -*ire* (ß = -2.41, SE = 0.24, z = -10.06, p < .0001), but there were no differences between -*are* and -*ire* (ß = 0.12, SE = 0.24, z = 0.50, p = .871).

In short, the results of Analysis 3 revealed that inflectional tasks were significantly easier than derivational tasks for both groups, although it should be observed that looking at raw data the difference between the two processes is quite small in terms of accuracy. Interestingly, dyslexics were overall less accurate than controls, independently of the type of process considered. Moreover, whereas controls had more difficulties with verbs in -*ere* but performed similarly with -*are* and -*ire*, dyslexics had the highest difficulties with -*ere* as well but were less accurate also with -*ire* as compared to -*are*.

### 3.5. Analysis 4

In our final analysis, we aimed at comparing the two groups in the two types of operation involved in our morphological tasks, namely verb production and verb base retrieval, in relation to the three conjugation classes. As shown in Table 9, dyslexics are always less accurate than controls, in both production and retrieval tasks; they are particularly inaccurate (only 0.06% of accuracy) in retrieving verb bases in -*ere*.

**Table 9:** Mean accuracy (SDs) of the two groups in tasks involving production and base retrieval in the three conjugation classes.

Group	Production -*are*	Production -*ere*	Production -*ire*	Base retrieval -*are*	Base retrieval -*ere*	Base retrieval -*ire*
**Con**	0.77 (0.41)	0.34 (0.47)	0.77 (0.42)	0.92 (0.27)	0.29 (0.53)	0.87 (0.33)
**Dys**	0.63 (0.48)	0.25 (0.43)	0.58 (0.50)	0.94 (0.26)	0.06 (0.24)	0.65 (0.48)

*Notes*. Con = Control Children; Dys = Dyslexic Children. The tasks considered are Tasks 2, 3, 4, 5 and 6 for Production and Tasks 8 and 10 for base retrieval.

To evaluate the performance of the two groups comparing the two types of morphological operations across the three Conjugation Classes, we ran a generalized linear mixed regression model with accuracy as dependent variable, adding Group (dyslexics vs. controls), Conjugation Class (-*are*, -*ere*, -*ire*) and Directionality of Operation (Production vs. Base Retrieval) as fixed effects with full interactions; Participant and Item were added as crossed random effects.

We found a significant main effect of Group, with dyslexics being less accurate than controls (β = -0.75, SE = 0.25, z = -3.00, p < .01), a main effect of Directionality of Operation, with accuracy being higher in tasks requiring base retrieval as compared to production (β = 1.38, SE = 0.43, z = 3.20, p < .01) and a main effect of Conjugation (χ^2^ = 176.30, df = 2, p < .0001); the interaction between Directionality of Operation and Conjugation was also significant (χ^2^ = 25.61, df = 2, p < .0001) as well as the interaction between Group, Directionality of Operation and Conjugation (χ^2^ = 11.56, df = 2, p < .01). The remaining interactions were not significant: Group*Directionality of Operation (χ^2^ = 2.79, df = 1, p = .095), Type of Process*Conjugation (χ^2^ = 3.82, df = 2, p = 0.15), and Group* Conjugation (χ^2^ = 4.45, df = 2, p = .108).

To investigate the nature of the Group*Directionality of Operation*Conjugation interaction, data were split according to both Group and Type of Operation and the effect of Conjugation was investigated in these four subsets running the relevant posthoc tests. As for dyslexics, in base retrieval verbs in -*ere* were more difficult to retrieve than verbs in -*are* (ß = -6.38, SE = 0.69, z = 9.28, p < .0001) and -*ire* (ß = -3.99, SE = 0.53, z = -7.48, p < .0001) and verbs in -*ire* more difficult than verbs in -*are* as well (ß = -2.39, SE = 0.46, z = -5.15, p < .0001). In production, instead, -*ere* was less correct than -*are* (ß = -2.87, SE = 0.33, z = 8.73, p < .0001) and -*ire* (ß = -2.15, SE = 0.32, z = -6.74, p < .0001), but the difference between -*ire* and -*are* was only marginally significant (ß = -0.72, SE = 0.31, z = -2.34, p = 0.51). As for control children, in base retrieval, verbs in -*ere* were more difficult than with verbs in -*are* (ß = -4.70, SE = 0.66, z = 7.15, p < .0001) and -*ire* (ß = -3.93, SE = 0.58, z = -6.76, p < .0001); no differences were instead found between -*ire* and -*are* (ß = -0.77, SE = 0.60, z = -1.29, p = .403). The same trend was found in production, where -*ere* was less accurate than -*are* (ß = -2.05, SE = 0.24, z = 8.52, p < .0001) and -*ire* (ß = -2.05, SE = 0.24, z = -8.52, p < .0001), with no differences between -*ire* and -*are* (ß = -0.01, SE = 2.44, z = -0.01, p = 1.00).

In summary, we found that, for both groups, tasks involving verb production were more difficult than tasks involving verb base retrieval, with dyslexics overall less accurate than controls. Moreover, looking at the three Conjugation Classes, as in Analysis 3 we found a significant difference between dyslexics and controls: while for controls only -*ere* was more difficult than -*are* and *-ire*, for dyslexics *-ire* was also more difficult than *-are*, although this difference was more marked in base retrieval tasks than in production tasks.

## 4. Discussion

In this study, we have presented the results of an original protocol aimed to provide an in-depth assessment of the morphological skills of children with dyslexia and typical development. Our protocol targeted both inflectional and derivational phenomena, and involved different lexical categories, in both production and base retrieval tasks. Although as reported in the introduction a growing interest has been recently observed in morphological development in dyslexia, studies have generally focused on specific aspects of morphology (especially, inflection) and on languages with deep orthographies, like English, whose inflectional morphology is also particularly poor. With our study, we aimed at casting further light on morphological awareness skills in dyslexia, exploring a wide range of processes and skills, and targeting Italian, a fusional language with shallow orthography and rich morphology, on which research in this domain is still limited.

Inspired by Berko’s original Wug test design and continuing a line of research commenced in previous studies [14, 64, 70], the 11 tasks in this protocol assessed children’s ability to manipulate nonwords. The use of nonwords allows one to disentangle vocabulary and morphological skills: the subjects’ morphological awareness, indeed, is measured by their ability to apply rules (or schemas) of inflection, derivation, and base retrieval to nonce words, which by definition cannot be part of their vocabulary. The design has been modeled according to the specific characteristics of the rich nominal and verbal paradigms of Italian, and especially focused on verb-based processes, by far less explored than those based on nouns.

Concerning **our first research aim**, i.e., that of investigating the morphological awareness of children with dyslexia, the results of Analysis 1 attest to a marked and generalized deficit in the dyslexic group, who showed an overall worse performance across the tasks compared to the group of age-matched typically developing children.

Children with dyslexia encountered difficulties in noun inflection, as indicated by the low scores obtained in noun pluralization (Task 1), confirming previous findings on this issue [14]. They did better, instead, in the derivation of evaluative nouns (Task 7), where their performance approached that of the control group, which was almost at ceiling (dyslexic children’s performance was inferior to that of control children, but the gap was only marginally significant). Compared to noun pluralization, children with dyslexia appeared less challenged by the core semantic properties of derivation, such as the formation of evaluatives, which are cognitively salient and very frequent in the input (evaluative forms of nouns and adjectives are used since children’s early development as they are characteristic of child-directed speech). Since Task 7 was the only task based on semantics instead of formal features, we hope to further test the semantics of derivation (and compounding) in future work, intending to get a deeper picture of the semantics of word formation in dyslexia.

With the other tasks, the research focus shifts to the verbal domain and, specifically, to verbal inflection and deverbal word formation. In this domain too, children with dyslexia had a significantly lower performance compared to their age-matched typically developing peers. The difficulties were especially found in verb inflection and in all base retrieval tasks (where children were asked to retrieve the target infinitive verb of an inflected or derived word form). Minor exceptions concerned two of the verb-based derivational tasks (i.e., in the derivation of deverbal nouns in -*mento* and adjectives in *-bile*), where dyslexia had only a marginally significant effect because the tasks were challenging for all children, as shown by the low accuracy scores.

Overall, the current study thus corroborates the results of previous research on Italian, which has already found an impairment in nonword pluralization in children with dyslexia [14]. However, as will be discussed in detail below, it also reveals that morphological awareness deficits in dyslexia are pervasive and go beyond the inflection of nouns, extending in particular to the verbal domain. Our findings also align with current research on other languages with rich morphology, where it was found that children with dyslexia underperform age-matched peers in inflection, derivation (see [52] on Bosnian) and compounding, too (see [54] on Greek).

Analysis 2 was specifically run to address the question of whether verb-based or noun-based morphology would be more challenging and/or specifically challenging for children with dyslexia, constituting our **second research aim**. In line with our predictions, all children had worse performances with verb-based than noun-based morphology: our verb-based tasks required the manipulation of subtle formal properties, involving the selection of specific vowels and implying a higher level of morphological awareness compared to noun pluralization and, especially, to the formation of evaluative nouns. In particular, the various degrees of transparency, regularity and frequency of verb-based processes affected the performance of all children in these tasks, with generally low performances in the most challenging conditions.

As for the **third research aim**, that of identifying and measuring the specific morphological domains in which children with dyslexia struggle the most, our results point to a generalized underperformance of the reading impaired children, whose morphological awareness deficits span across types of tasks (i.e., inflection and derivation) and directionality of the operation (i.e., production of a complex form and retrieval of the base). Moreover, as will be discussed below in greater detail, conditions (in particular, conjugation classes) also feature as a prominent factor, with effects on accuracy across tasks in both groups of children.

More specifically, Analysis 3 explored the potential effects of type of process (infection vs. derivation) and dyslexia on accuracy. Children with dyslexia were found significantly challenged by both types of tasks, underperforming the control group, with accuracy being lower in derivational than in inflection tasks. Moreover, the absence of the interaction between group and type of process reveals that *all* children encountered more troubles in derivation, especially derivational processes requiring the manipulation of markers of conjugation classes, than in inflectional phenomena requiring the identification of declension and conjugation classes. It should be observed that in terms of raw percentages accuracy is only slightly lower in derivational with respect to inflectional tasks; yet the difference is statistically significant. Although more research is thus needed to assess the difference between the two types of processes, this preliminary result is in line with previous findings on the development of morphology, at both the epilinguistic and metalinguistic levels: being more systematic and frequent, inflection starts to be learned very early on, while derivational rules, being semantically complex and less regular, are learned later in language development [19]; similarly, awareness of derivation tends to emerge later than awareness of inflection [10]. The lack of interaction between group and type of process also shows that this holds across children, independently of dyslexia.

Analysis 4, instead, assessed the potential effects of the directionality of the operation on children’s accuracy, also considering possible differences between groups: is it easier to produce a complex word, either inflected or derived, starting from the infinitival base, or to retrieve the base form of the verb from a complex word? Does dyslexia worsen performance in these types of tasks? Starting from the latter question, both production and base retrieval let emerge significant differences between groups, with dyslexic children being less accurate than controls in both types of tasks. However, whilst typically developing children exhibited an overall good performance in base retrieval, measuring the subject’s ability to identify and produce pseudo-infinitives from morphologically complex pseudo-words, children with dyslexia were particularly less accurate, with results close to zero in the most challenging condition.

Although children with dyslexia underperformed typically developing children, results showed that all participants were generally more accurate in base retrieval, where they had to retrieve an infinitive from an inflected or derived verb, than in production tasks, where they had to produce an inflected or derived form starting from an infinitive. This finding can be explained by the fact that production tasks tap more directly (unconscious) morphological processing, since they more naturally mimic what happens in a natural speech setting, where speakers form complex words by combining affixes and bases, i.e., through an additive operation. Interestingly, indeed, all children were more prone to mistakes of overregularization in production tasks: i.e., they often overextended the most common theme vowel (-*a*) to verbal stems of the other conjugation classes. Typical mistakes across subjects were, in past participle formation along the lines of *nov-ere* > *nov-a-to instead of *nov-u-to* and, in deverbal nominalization, *vud-ire* > *vud-a-mento instead of *vud-i-mento*. However, in some cases children failed to identify the base completely, attaching the suffix to a (non-target) base containing the *-r-* that is part of the inflectional suffix given in the elicitation formula (like in *mann-ire* > *mannir-ato instead of *mann-ito*) or preserving the *-e-* in Conj. II verb-based formations (like in *cuv-ere* > *cuv-e-mento instead of *cuv-i-mento*). In other cases, they failed in the selection of the theme vowel preceding the suffix, extending -*i*-, which was more frequent in the stimuli, to the most default class in -*a*- (like in base retrieval from *pam-a-ta* > *pam-ire instead of *pam-are*). Overall, it looks like this type of process, though more natural and frequent, led more easily to inaccurate outputs, which is arguably due to lesser morphological awareness (especially, yet not limited to, derivation). On the other hand, retrieval tasks, though letting emerge drastic differences between conditions in children with dyslexia, triggered overall higher accuracy rates across our subjects. In fact, like nonword production, retrieval also implies a high level of morphological awareness since it requires the metalinguistic ability to identify the verbal root and select the target infinitive. However, verb base retrieval is based on a type of knowledge that is also more explicitly taught in class, since the study of verbal conjugations and the identification of theme vowels (especially starting from inflected forms) is part of standard education programs in Italian primary schools. Therefore, the more conscious reflection upon word structure, strengthened by the awareness acquired in school, may have supported children in retrieval tasks compared to production tasks. Notice moreover that the interaction that we found between group, directionality of the process, and conjugation class showed that while for controls only -*ere* was more difficult than -*are* and *-ire* and no differences were found between *-are* and *-ire*, a different pattern is observed in dyslexics, where *-ire* was also more difficult than *-are* in the base retrieval tasks (only a marginal significance was found in production tasks). This seems to indicate that for dyslexics the higher cost in terms of morphological awareness required by the base retrieval tasks had a negative effect not only on the least regular, transparent, and productive conjugation class (Conj. II, -*ere* verbs) but also on the verbs in -*ire*, belonging to Conj. III, whose transparency, regularity, and productivity are higher than those of Conj. II, but still lower than those of Conj. I (-*are* verbs).

This leads us to the focus of our **fourth research aim**, precisely concerning the assessment of the effects of transparency, frequency, productivity, and regularity across the experimental conditions. We analyzed these effects by including the experimental condition (in terms of different conjugation classes) as a fixed effect in Analyses 3 and 4, intending to measure its influence on accuracy in interaction with group, type of process (Analysis 3), and directionality of the operation (Analysis 4). The results of the statistical analyses, overall, confirmed our expectations, since all children tendentially scored better in the experimental conditions built on the most transparent, frequent, productive, and regular declension and conjugation classes. On the other hand, the least transparent, productive, and regular conjugation class (Conj. II) was the most challenging for everyone. This pattern of results applied across inflection and derivation, and also across production and base retrieval tasks, showing the consistent effect of variables such as transparency, frequency, and productivity of formal features in the phenomena under investigation.

Let us consider inflection first. For instance, in the formation of past participles, all children were particularly challenged by pseudo-verbs of the -*ere* class (Conj. II), arguably for reasons related to their mid-low frequency in the Italian lexicon and their lesser regularity and transparency. Indeed, -*ere* verbs are characterized by different degrees of allomorphy in their past participle stems, e.g., *bere* ‘to drink’ > *bev-uto* ‘drunk’ vs. *correre* ‘to run’ > *cor-s-o ‘*run’ vs. *vedere* ‘to see’ *> visto* ‘seen’; this allomorphy hinders the acquisition and processing of the past participles in -*u-to*, which even in the regular cases involve a vowel, -*u*-, which is not part of the infinitival stem and weakens the formal transparency of the inflected form. Unsurprisingly, a similar pattern emerged in the derivation tasks: all children performed better with the most productive, formally transparent, and regular verbs in -*are* (Conj. I), whilst the derivation or retrieval of verbs in -*ere* was the most challenging.

However, our results also revealed interesting differences between children with dyslexia and control children concerning the -*ire* class (Conj. III): as predicted, due to their mild productivity and higher transparency, -*ire* verbs were less problematic than -*ere* verbs, but children with dyslexia were significantly less accurate with the verbal forms in -*ire* than with those in -*are*. This pattern was not found with control children, who were comparatively less challenged by this class, with no significant difference between -*are* and -*ire* verbs. This result indicates that children with dyslexia show marked weaknesses also with a class that, notwithstanding its lesser frequency, is mildly productive and regular, showing therefore a lower level of awareness of their rich morphological system compared to the typically developing peers.

A similar result was found in Analysis 4, which considered the role of conjugation classes across tasks of production and base retrieval. Here we found again the same pattern, with -*ere* verbs being the most challenging and -*are* the least problematic for all children. It must be noted, moreover, that dyslexia seems to especially worsen the performance with the retrieval of pseudo-verbs in -*ere*, where the reading-impaired children had a score close to zero, showing a complete inability to retrieve the base verbs from the most irregular and unproductive conjugation class. However, typically developing and dyslexic children’s performance was differently affected by -*ire* verbs in base retrieval tasks: while control children showed no significant differences in base retrieval from -*are* and -*ire* verbs, here again, the retrieval of -*ire* verbs was more difficult than that of -*are* verbs, singling out the specific weaknesses of children with dyslexia. Therefore, children with dyslexia seemed overall highly challenged by retrieval tasks, requiring more explicit reflection upon word structure, and they most often retrieved nonce verbs of the first conjugation in all conditions, manifesting scarce awareness of conjugation markers.

In summary, our study reveals that children with dyslexia experience difficulties that span across various domains of morphological awareness, i.e., inflection vs. derivation, production vs. base retrieval, and across different lexical categories and conjugation classes (corresponding to the conditions of our tasks). Like control children, children with dyslexia were especially challenged by derivation tasks and production tasks, especially in conditions replicating less frequent and non-transparent word formation processes. However, a gap with typically developing children’s performance was found in tasks and conditions that emerged as not especially challenging for the control children. This attests to a lower level of morphological awareness in children with dyslexia, who only master the most transparent, frequent, and regular operations but are challenged by those operations that manifest mid to low degrees of transparency, frequency, and regularity.

The results of this study are in line with previous research on morphological awareness in dyslexia, which has mainly focused on its relation to reading achievements in reading-impaired populations. This is a topic only marginally touched by the current study, whose scope was broad, yet confined to morphological abilities. We aim to develop a broader assessment of language abilities (especially, including measures of phonological awareness) and reading skills in future work, which will allow us to measure the (unique) contribution of morphological awareness to reading achievements in dyslexia, along the lines of our previous studies [14]. Furthermore, the current research was mainly limited to formal features of word formation. As discussed above, semantics was only specifically tapped by Task 7, assessing the formation of evaluative nouns, which is a rather basic derivational process (as suggested by the ceiling performance of the control group). In this task, too, children with dyslexia emerged as less skilled than their peers, although the group difference was only marginally significant. Therefore, future research could further test children with dyslexia on the semantics of word formation and explore its relation to reading achievements (both decoding and comprehension skills).

Finally, whilst it is a fact that morphological awareness is hampered by dyslexia across different languages, we do not expect our results to necessarily hold crosslinguistic validity. On the contrary, we would like to emphasize that the results we got in this study are tied to the features of Italian morphology and, as such, they cannot define morphological awareness in children with dyslexia in a broad sense. Paraphrasing [80: 149] words about children with DLD, *children with developmental dyslexia look first and foremost like speakers and readers of the type of language to which they are exposed*, *and only secondarily like poor speakers and readers of that language*. In layman’s words, the fine-grained language profile and, more specifically, the morphological awareness profile of a child with dyslexia is crucially determined by the specificities and challenges of the language system in use, which are not generalizable to all languages. Nonetheless, it would be extremely interesting to identify consistent tendencies across languages, like effects of frequency, productivity, regularity, and transparency of the morphological phenomena, or of the directionality of the operation, along the lines of our study. This would help the scientific community get a more profound understanding of the morphological awareness profile of children with dyslexia and typical development from a crosslinguistic perspective, with possibly relevant implications for the less explored or resourced languages, and especially to provide specific indications for more appropriate teaching and intervention programs.

## 5. Conclusion

This study has brought fresh empirical evidence on the presence and extent of morphological impairments in dyslexic children. These deficits affect inflectional and derivational morphology, span over lexical categories and declension/conjugation classes, and hamper the dyslexic children’s ability to extract and apply patterns of inflection and word formation. Therefore, our results confirm the characterization of dyslexia as an impairment that extends beyond phonology and interferes with the development of morphological awareness, in line with recent research on other languages.

As reviewed in the introduction, research conducted on individuals with reading disorders across different ages has established that morphological awareness skills increasingly assume a key role in literacy and emerge as a compensatory tool for the major phonological deficits characterizing dyslexia in the course of literacy development. The presence of a morphological impairment in children with dyslexia, combined with the crucial role of morphology for reading and spelling skills in impaired readers, can have educational and clinical implications, indicating the potential relevance of reinforcing morphological awareness, especially in the case of developmental dyslexia.

Dyslexic children, who suffer from a phonological impairment that persists in adulthood, could greatly benefit from education programs explicitly focusing on word structure and from speech therapy aimed at enhancing their morphological abilities. In particular, teaching programs could insist on methods for reinforcing awareness of inflection and, especially, derivation, a domain that is comparatively more neglected in standard education programs. The deployment of nonce words, besides standard practice with real words, could also help emphasize formal and semantic aspects of word formation that often go unnoticed, with the (possible) effect of raising the morphological awareness of all children. Speech therapy could also incorporate the results of current research on morphology and devise pilot training programs aimed at enhancing morphological awareness, besides other sublexical knowledge (i.e., syllables and phonemes). Specifically, the training could be aimed at reinforcing the domains where children with dyslexia struggle the most, based on our findings and other research. Indeed, a more efficient recognition of morphemic units in written words, and of form/meaning relations between morphological families and series of words, could aid dyslexic children to achieve faster and more accurate decoding abilities, while also improving their comprehension skills. Moreover, as shown by previous results [5, 21], the cognizance of morphemes as minimal meaningful units and the metalinguistic ability to decompose novel words into meaningful parts seem to play a crucial role in word and text comprehension. Nevertheless, notice that, with a view to inclusion, this kind of activity could be particularly useful to increase morphological awareness, alongside reading and comprehension skills, not only for children with dyslexia but also for children with language vulnerabilities (as second language learners) and with typical development. We leave the exploration of this relevant aspect of morphological awareness for future research.

## References

[1] Carlisle JF. Morphological Awareness and Early Reading Achievement. In: Feldman LB, editor. Morphological aspects of language processing, Hillsdale, NJ: Erlbaum; 1995, p. 189–209.

[2] Carlisle JF. Awareness of the structure and meaning of morphologically complex words: Impact on reading. Reading and Writing 2000;12:169–90. 10.1023/A:1008131926604.

[3] Carlisle JF, Stone CA. Exploring the role of morphemes in word reading. Reading Research Quarterly 2005;40:428–49. 10.1598/RRQ.40.4.3.

[4] Deacon SH, Kirby JR. Morphological awareness: Just “more phonological”? The roles of morphological and phonological awareness in reading development. Applied Psycholinguistics 2004;25:223–38. 10.1017/S0142716404001110.

[5] Elbro C, Arnbak E. The role of morpheme recognition and morphological awareness in dyslexia. Annals of Dyslexia 1996;46:209–40. doi: 10.1007/BF02648177 24234273

[6] Kemp N. Children’s spelling of base, inflected, and derived words: Links with morphological awareness. Reading and Writing: An Interdisciplinary Journal 2006;19:737–65. 10.1007/s11145-006-9001-6.

[7] Nagy W, Berninger VW, Abbott RD. Contributions of morphology beyond phonology to literacy outcomes of upper elementary and middle-school students. Journal of Educational Psychology 2006;98:134–47. 10.1037/0022-0663.98.1.134.

[8] Singson M, Mahony D, Mann V. The relation between reading ability and morphological skills: Evidence from derivational suffixes. Reading and Writing 2000;12:219–52. 10.1023/A:1008196330239.

[9] Freitas PVD, Mota MMPED, Deacon SH. Morphological awareness, word reading, and reading comprehension in Portuguese. Applied Psycholinguistics 2018;39:507–25. 10.1017/S0142716417000479.

[10] Diamanti V, Benaki A, Mouzaki A, Ralli A, Antoniou F, Papaioannou S, et al. Development of early morphological awareness in Greek: Epilinguistic versus metalinguistic and inflectional versus derivational awareness. Applied Psycholinguistics 2018;39:545–67. 10.1017/S0142716417000522.

[11] Marcolini S, Traficante D, Zoccolotti P, Burani C. Word frequency modulates morpheme-based reading in poor and skilled Italian readers. Applied Psycholinguistics 2011;32:513–32. 10.1017/S0142716411000191.

[12] Vender M. Disentangling Dyslexia. Phonological and Processing Impairment in Developmental Dyslexia. Bern: Peter Lang; 2017. 10.3726/b11503.

[13] Joanisse MF, Manis FR, Keating P, Seidenberg MS. Language deficits in dyslexic children: speech perception, phonology, and morphology. J Exp Child Psychol 2000;77:30–60. doi: 10.1006/jecp.1999.2553 10964458

[14] Vender M, Mantione F, Savazzi S, Delfitto D, Melloni C. Inflectional morphology and dyslexia: Italian children’s performance in a nonword pluralization task. Ann of Dyslexia 2017;67:401–26. 10.1007/s11881-017-0152-8. 29134481

[15] Burani C, Marcolini S, Traficante D, Zoccolotti P. Reading Derived Words by Italian Children With and Without Dyslexia: The Effect of Root Length. Front Psychol 2018;9:647. doi: 10.3389/fpsyg.2018.00647 29867633 PMC5952107

[16] Burani C. Word morphology enhances reading fluency in children with developmental dyslexia. Lingue e Linguaggio 2010;9:177–98.

[17] Clark EV. The young word maker: A case study of innovation in the child’s lexicon. In: Wanner E, Gleitman L, editors. Language acquisition: The state of the art, Cambridge: Cambridge University Press; 1982, p. 390–425.

[18] Nagy WE, Diakidoy IAN, Anderson RC. The acquisition of morphology: Learning the contribution of suffixes to the meanings of derivatives. Journal of Literacy Research 1993;25:155–70. 10.1080/10862969309547808.

[19] Clark EV. Morphology in Language Acquisition. In: Spencer A, Swicky AM, editors. The Handbook of Morphology, Oxford: Blackwell; 1998, p. 374–89.

[20] Ivanova-Sullivan T, Sekerina IA. Distributional Regularity of Cues Facilitates Gender Acquisition: A Contrastive Study of Two Closely Related Languages. In: Brown MM, Dailey B, editors. Proceedings of the 43rd Boston University Conference on Language Development, Somerville, MA: Cascadilla Press; n.d., p. 311–23.

[21] Casalis S, Colé P, Sopo D. Morphological awareness in developmental dyslexia. Ann of Dyslexia 2004;54:114–38. doi: 10.1007/s11881-004-0006-z 15765006

[22] Gombert JE. Metalinguistic development. Chicago: University of Chicago Press; 1992.

[23] Duncan LG, Seymour PH, Hill S. A small-to-large unit progression in metaphonological awareness and reading? Q J Exp Psychol A 2000;53:1081–104. doi: 10.1080/713755936 11131814

[24] Berko J. The Child’s Learning of English Morphology. Word 1958;14:150–77. 10.1080/00437956.1958.11659661.

[25] Carlisle JF, Nomanbhoy DM. Phonological and morphological awareness in first graders. Applied Psycholinguistics 1993;14:177–95. 10.1017/S0142716400009541.

[26] Cunningham AJ, Carroll JM. Early predictors of phonological and morphological awareness and the link with reading: Evidence from children with different patterns of early deficit. Applied Psycholinguistics 2015;36:509–31. 10.1017/S0142716413000295.

[27] Kieffer MJ, Lesaux NK. Development of morphological awareness and vocabulary knowledge in Spanish-speaking language minority learners: A parallel process latent growth curve model. Applied Psycholinguistics 2012;33:23–54. 10.1017/S0142716411000099.

[28] Apel K, Lawrence J. Contributions of morphological awareness skills to word-level reading and spelling in first-grade children with and without speech sound disorder. Journal of Speech, Language, and Hearing Research 2011;54:1312–27. doi: 10.1044/1092-4388(2011/10-0115) 21386040

[29] Reed DK. A Synthesis of Morphology Interventions and Effects on Reading Outcomes for Students in Grades K–12. Learning Disabil Res Pract 2008;23:36–49. 10.1111/j.1540-5826.2007.00261.x.

[30] Carlisle JF, McBride-Chang C, Nagy W, Nunes T. Effects of Instruction in Morphological Awareness on Literacy Achievement: An Integrative Review. Reading Research Quarterly 2010;45:464–87. 10.1598/RRQ.45.4.5.

[31] Bowers PN, Kirby JR, Deacon SH. The Effects of Morphological Instruction on Literacy Skills: A Systematic Review of the Literature. Review of Educational Research 2010;80:144–79. 10.3102/0034654309359353.

[32] Goodwin AP, Ahn S. A meta-analysis of morphological interventions: effects on literacy achievement of children with literacy difficulties. Ann Dyslexia 2010;60:183–208. doi: 10.1007/s11881-010-0041-x 20799003

[33] American Psychiatric Association. Diagnostic and Statistical Manual of Mental Disorders. Fifth Edition. American Psychiatric Association; 2013. 10.1176/appi.books.9780890425596.

[34] Snowling MJ. Phonological processing and developmental dyslexia. Journal of Research in Reading 1995;18:132–8. 10.1111/j.1467-9817.1995.tb00079.x.

[35] Ramus F, Szenkovits G. What Phonological Deficit? Quarterly Journal of Experimental Psychology 2008;61:129–41. doi: 10.1080/17470210701508822 18038344

[36] Desroches AS, Joanisse MF, Robertson EK. Specific phonological impairments in dyslexia revealed by eyetracking. Cognition 2006;100:B32–42. doi: 10.1016/j.cognition.2005.09.001 16288732

[37] Melloni C, Vender M. Phonological Processing and Nonword Repetition: A Critical Tool for the Identification of Dyslexia in Bilingualism. In: Babatsouli E, Ball MJ, editors. Anthology of bilingual child phonology, Multilingual Matters; 2020, p. 309–33. 10.21832/9781788928427-015.

[38] Vender M, Delfitto D, Melloni C. How do bilingual dyslexic and typically developing children perform in nonword repetition? Evidence from a study on Italian L2 children. Bilingualism 2020;23:884–96. 10.1017/S1366728919000828.

[39] Vender M, Melloni C. Phonological Awareness across Child Populations: How Bilingualism and Dyslexia Interact. Languages 2021;6:39. 10.3390/languages6010039.

[40] Scarborough HS. Very Early Language Deficits in Dyslexic Children. Child Development 1990;61:1728–43. 10.1111/j.1467-8624.1990.tb03562.x. 2083495

[41] Lyytinen P, Poikkeus A-M, Laakso M-L, Eklund K, Lyytinen H. Language Development and Symbolic Play in Children With and Without Familial Risk for Dyslexia. Journal of Speech, Language, and Hearing Research 2001;44:873–85. doi: 10.1044/1092-4388(2001/070) 11521780

[42] Snowling MJ, Gallagher A, Frith U. Family Risk of Dyslexia Is Continuous: Individual Differences in the Precursors of Reading Skill. Child Development 2003;74:358–73. doi: 10.1111/1467-8624.7402003 12705560

[43] Bar-Shalom EG, Crain S, Shankweiler D. A comparison of comprehension and production abilities of good and poor readers. Applied Psycholinguistics 1993;14:197–227. 10.1017/S0142716400009553.

[44] Byrne B. Deficient syntactic control in poor readers: Is a weak phonetic memory code responsible? Applied Psycholinguistics 1981;2:201–12. 10.1017/S0142716400006512.

[45] Robertson EK, Joanisse M. Spoken sentence comprehension in children with dyslexia and language impairment: The roles of syntax and working memory. Applied Psycholinguistics 2009. 10.1017/S0142716409990208.

[46] Vender M, Hu S, Mantione F, Delfitto D, Melloni C. The Production of Clitic Pronouns: A Study on Bilingual and Monolingual Dyslexic Children. Front Psychol 2018;9:2301. doi: 10.3389/fpsyg.2018.02301 30542308 PMC6277746

[47] Waltzman DE, Cairns HS. Grammatical knowledge of third grade good and poor readers. Applied Psycholinguistics 2000;21:263–84. 10.1017/S014271640000206X.

[48] Jeffries S, Everatt J. Working memory: Its role in dyslexia and other specific learning difficulties. Dyslexia 2004;10:196–214. doi: 10.1002/dys.278 15341198

[49] Jimenez JE, Garcia E, Estevez A, Diaz A, Guzman R, Hernandez-Valle I, et al. An Evaluation of Syntactic-Semantic Processing in Developmental Dyslexia. Electronic Journal of Research in Educational Psychology 2004;2:127–42.

[50] Rispens JE, McBride-Chang C, Reitsma P. Morphological awareness and early and advanced word recognition and spelling in Dutch. Read Writ 2008;21:587–607. doi: 10.1007/s11145-007-9077-7

[51] Deacon H, Tong SX, Mimeau C. Morphological and semantic skills in developmental dyslexia across languages. In: Perfetti C, Pugh K, Verhoeven L, editors. Dyslexia across languages and writing systems: A handbook., Cambridge: Cambridge University Press; 2016.

[52] Duranovic M, Tinjak S, Turbic-Hadzagic A. Morphological Knowledge in Children with Dyslexia. J Psycholinguist Res 2014;43:699–713. doi: 10.1007/s10936-013-9274-2 24197938

[53] Rothou KM, Padeliadu S. Morphological processing influences on dyslexia in Greek-speaking children. Ann Dyslexia 2019;69:261–78. doi: 10.1007/s11881-019-00184-8 31529233

[54] Giazitzidou S, Padeliadu S. Contribution of morphological awareness to reading fluency of children with and without dyslexia: evidence from a transparent orthography. Ann Dyslexia 2022. doi: 10.1007/s11881-022-00267-z 35907104

[55] Traficante D, Marcolini S, Luci A, Zoccolotti P, Burani C. How do roots and suffixes influence reading of morphological pseudowords: A study of Italian children with dyslexia. Language and Cognitive Processes 2011;26:777–93. 10.1080/01690965.2010.496553.

[56] Burani C, Marcolini S, De Luca M, Zoccolotti P. Morpheme-based reading aloud: Evidence from dyslexic and skilled Italian readers. Cognition 2008;108:243–62. doi: 10.1016/j.cognition.2007.12.010 18262178

[57] Arnbak E, Elbro C. The Effects of Morphological Awareness Training on the Reading and Spelling Skills of Young Dyslexics. Scandinavian Journal of Educational Research 2000;44:229–51. 10.1080/00313830050154485.

[58] Cavalli E, Duncan LG, Elbro C, El Ahmadi A, Colé P. Phonemic—Morphemic dissociation in university students with dyslexia: an index of reading compensation? Ann of Dyslexia 2017;67:63–84. doi: 10.1007/s11881-016-0138-y 27739013

[59] Martin J, Frauenfelder UH, Colé P. Morphological awareness in dyslexic university students. Applied Psycholinguistics 2014;35:1213–33. 10.1017/S0142716413000167.

[60] Law JM, Wouters J, Ghesquière P. Morphological Awareness and Its Role in Compensation in Adults with Dyslexia. Dyslexia 2015;21:254–72. doi: 10.1002/dys.1495 25620091

[61] Pescuma VN, Zanini C, Crepaldi D, Franzon F. Form and Function: A Study on the Distribution of the Inflectional Endings in Italian Nouns and Adjectives. Frontiers in Psychology 2021;12. 10.3389/fpsyg.2021.720228 PMC8529016 34690878

[62] Dressler W, Thornton AM. Italian nominal inflection. Wiener Linguistische Gazette 1996;57–59:1–26.

[63] Acquaviva P. The Structure of the Italian Declension System. In: Montermini F, Boyé G, Tseng J, editors. Selected Proceedings of the 6th Décembrettes, Somerville, MA: Cascadilla Proceedings Project; 2009, p. 50–62.

[64] Vender M, Hu S, Mantione F, Savazzi S, Delfitto D, Melloni C. Inflectional morphology: evidence for an advantage of bilingualism in dyslexia. International Journal of Bilingual Education and Bilingualism 2021;24:155–72. 10.1080/13670050.2018.1450355.

[65] Pirrelli V, Battista M. The paradigmatic dimension of stem allomorphy in Italian verb inflection: 2628. Italian Journal of Linguistics 2000;12:307–80.

[66] Napoli DJ, Vogel I. The conjugations of Italian. Italica 1990;67:479–502.

[67] Orsolini M, Fanari R, Bowles H. Acquiring regular and irregular inflection in a language with verb classes. Language and Cognitive Processes 1998;13:425–64. 10.1080/016909698386456.

[68] Eddington D. Dissociation in Italian Conjugations: A Single-Route Account. Brain and Language 2002;81:291–302. 10.1006/brln.2001.2525. 12081400

[69] Say T, Clahsen H. Words, rules and stems in the Italian mental lexicon. In: Nooteboom S, Weerman, Wijnen F, editors. Words, rules and stems in the Italian mental lexicon, Dordrecht: Kluwer Academic Publishers; 2002, p. 93–129.

[70] Melloni C, Vender M, Delfitto D. Inflectional morphology: evidence for an advantage of bilingualism in Albanian-Italian and Romanian-Italian bilingual children. In: Slabakova R, Corbet J, Dominguez L, Dudley A, Wallington A, editors. Explorations in Second Language Acquisition and Processing, New Castle: Cambridge Scholars Publishing; 2019, p. 238–50.

[71] Belacchi C, Scalisi TG, Cannoni E, Cornoldi C. CPM–Coloured Progressive Matrices. Standardizzazione italiana. Florence, Italy: Giunti OS, Organizzazioni Speciali; 2008.

[72] Dunn LM, Dunn LM. PEABODY—Test di Vocabolario Recettivo—P.P.V.T.-R. Peabody Picture Vocabulary Test—Revised—Test Psicolinguistico. Adattamento italiano e standardizzazione a cura di: Giacomo Stella—Claudia Pizzioli—Patrizio E. Tressoldi. Torino: Omega Edizioni; 2000.

[73] Sartori G, Job R, Tressoldi PE. DDE-2. Batteria per la Valutazione della Dislessia e della Disortografia Evolutiva-2. Firenze: Giunti OS, Organizzazioni Speciali; 2007.

[74] Stella V, Job R. Le sillabe PD/DPSS. Una base di dati sulla frequenza dell’italiano scritto. Giornale Italiano Di Psicologia 2001;3:633–9.

[75] Guasch M, Boada R, Ferré P, Sánchez-Casas R. NIM: A web-based Swiss army knife to select stimuli for psycholinguistic studies. Behavior Research Methods 2013;45:765–71. doi: 10.3758/s13428-012-0296-8 23271155

[76] R Core Team. R: A language and environment for statistical ## computing. R Foundation for Statistical Computing, Vienna, Austria. 2022.

[77] Bates D, Mächler M, Bolker B, Walker S. Fitting Linear Mixed-Effects Models Using lme4. Journal of Statistical Software 2015;67:1–48. 10.18637/jss.v067.i01.

[78] Kuznetsova A, Brockhoff PB, Christensen RHB. lmerTest Package: Tests in Linear Mixed Effects Models. Journal of Statistical Software 2017;82:1–26. 10.18637/jss.v082.i13.

[79] Lenth R. emmeans: Estimated Marginal Means, aka Least-Squares Means 2022.

[80] Leonard LB. Children with specific language impairment. Second edition. Cambridge, Massachusetts: The MIT Press; 2014.

